# Mapping cell surface adhesion by rotation tracking and adhesion footprinting

**DOI:** 10.1038/srep44502

**Published:** 2017-03-14

**Authors:** Isaac T. S. Li, Taekjip Ha, Yann R. Chemla

**Affiliations:** 1 Department of Physics and Center for Physics of Living Cells, University of Illinois at Urbana-Champaign, 1110 W Green St., Urbana, IL, 61801, USA; 2 Howard Hughes Medical Institute, Department of Biophysics and Biophysical Chemistry, Department of Biophysics and Department of Biomedical Engineering, Johns Hopkins University, 725 N. Wolfe Street, Baltimore, MD, 21205, USA

## Abstract

Rolling adhesion, in which cells passively roll along surfaces under shear flow, is a critical process involved in inflammatory responses and cancer metastasis. Surface adhesion properties regulated by adhesion receptors and membrane tethers are critical in understanding cell rolling behavior. Locally, adhesion molecules are distributed at the tips of membrane tethers. However, how functional adhesion properties are globally distributed on the individual cell’s surface is unknown. Here, we developed a label-free technique to determine the spatial distribution of adhesive properties on rolling cell surfaces. Using dark-field imaging and particle tracking, we extract the rotational motion of individual rolling cells. The rotational information allows us to construct an adhesion map along the contact circumference of a single cell. To complement this approach, we also developed a fluorescent adhesion footprint assay to record the molecular adhesion events from cell rolling. Applying the combination of the two methods on human promyelocytic leukemia cells, our results surprisingly reveal that adhesion is non-uniformly distributed in patches on the cell surfaces. Our label-free adhesion mapping methods are applicable to the variety of cell types that undergo rolling adhesion and provide a quantitative picture of cell surface adhesion at the functional and molecular level.

Rolling adhesion is a common process by which cells attach themselves to surfaces under shear flow, such as in the circulatory system. Leukocytes in the blood utilize this mechanism to locate inflammation sites throughout the body. During an inflammation response, endothelial cells lining the blood vessels surrounding an infection site express adhesion proteins called selectins that are specific to leukocyte surface receptors. As the first step of the leukocyte adhesion cascade, leukocytes captured via selectin-specific interactions passively roll on the blood vessel wall under blood flow toward the inflammation site in a process known as rolling adhesion^1^,^2^,^3^. Malfunction of any adhesion molecules involved in this process leads to severe immune disorders such as the leukocyte adhesion deficiencies (LAD)^4^. Rolling adhesion behavior is also exhibited by circulating tumor cells (CTCs) which is believed to enhance cancer metastasis^5^,^6^,^7^,^8^. Therefore, quantitative understanding of rolling adhesion is necessary to enable practical applications such as cancer screening and treatment^9^,^10^,^11^.

At the molecular level, this adhesion is mediated by catch-bond-like interactions^12^,^13^ between P-14 and E-selectins^15^ expressed on endothelial cells lining blood vessels and P-selectin glycoprotein ligand-1 (PSGL-1) found at microvilli tips of leukocytes^16^. Despite our understanding of the individual components, how the molecular details of adhesion bonds scale to cell-surface adhesion and rolling behavior remains poorly understood^2^,^17^,^18^.

Here, we developed a label-free method that maps the functional adhesion sites and strengths on a cell surface as it rolls across a surface coated uniformly with adhesion receptors. The method relies on tracking the rotational angle of a single rolling cell, which confers advantages over standard methods that track the center-of-mass alone^19^. Constructing the adhesion map from the instantaneous angular velocity reveals that the adhesion profile along the rolling circumference is inhomogeneous. We corroborated these findings by obtaining fluorescent footprints of molecular adhesion events using probes derived from recently developed DNA-based molecular force sensors^20^. Our results reveal that adhesion at the functional level is not uniformly distributed over rolling cell surface as previously assumed^21^, but is instead patchy. Our methods will enable researchers to generate significantly richer data when studying the rolling adhesion of immune cells and circulating tumor cells.

## Results

### Rotation tracking of rolling motion

**Figure 1:**
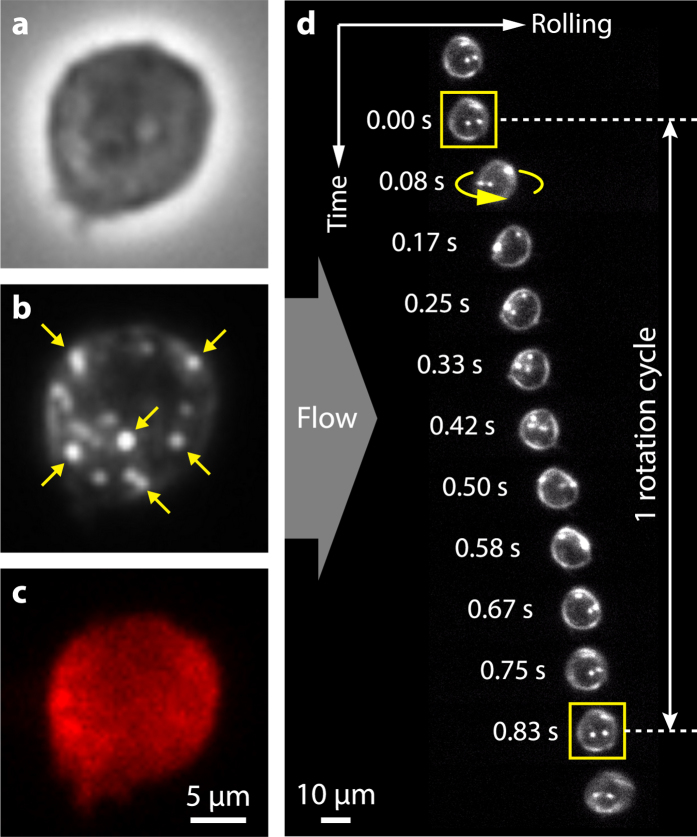
Dark-field microscopy reveals intracellular markers for rotation tracking. Images of the same cell at 60x magnification by: (**a**) phase-contrast imaging, (**b**) dark-field microscopy, with arrows indicating intracellular granules, (**c**) Cy5 fluorescence imaging of DiD-labeled membranes. (**d**) Snapshots of a representative rolling cell with time stamps (~0.08 s interval) on a P-selectin coated surface at 25x magnification with flow direction pointing to the right. The rolling cell is under a constant shear stress of 1.2 Pa. Two bright spots inside the cell serve as visual reference markers to track the cell rotation. Initially, the two visible spots are at the bottom surface of the cell. As the cell rolls in the direction of the arrow, the spots rotate to the top surface and back to the bottom again. Two yellow boxes mark ~1 rotation cycle of the cell with the spots returning to the same position.

Mapping rolling cell surface adhesion properties requires knowing at all times which point(s) on the cell contact the surface on which it rolls. Unfortunately, tracking the translation of the cell’s center of mass, as done in most conventional cell rolling assays^19^, does not provide a direct measurement of the surface contact point. In principle, it is possible to access this information by tracking the cell’s orientation as it rolls. In order to measure the rotation of a rolling cell, we developed a method that tracks intracellular reference markers that rotate with the cell. We used the HL-60 (Human promyelocytic leukemia cells) cell line as a model^11^,^22^,^23^ for rolling adhesion (Materials and Methods). Unlike phase-contrast or fluorescence imaging, which have typically been used for whole cell identification and tracking^19^,^24^ (Fig. 1a,b), dark-field microscopy reveals μm to sub-μm-sized, highly scattering spots inside most HL-60 cells (Fig. 1c). We speculate that these bright spots in HL-60 cells are azurophilic granules^25^, which are denser (1.1 g/mL) and scatter more light than other organelles. The spots can be visually separated from one another due to their low copy number and high contrast against the background, and they are approximately immobile over the course of a typical experiment (~30 s) (Supp. Figure S1). In Fig. 1d, an HL-60 cell imaged with dark-field microscopy is shown rolling on a P-selectin-coated surface (Materials and Methods). The spot pattern clearly repeats as the cell completes each rolling cycle.

**Figure 2:**
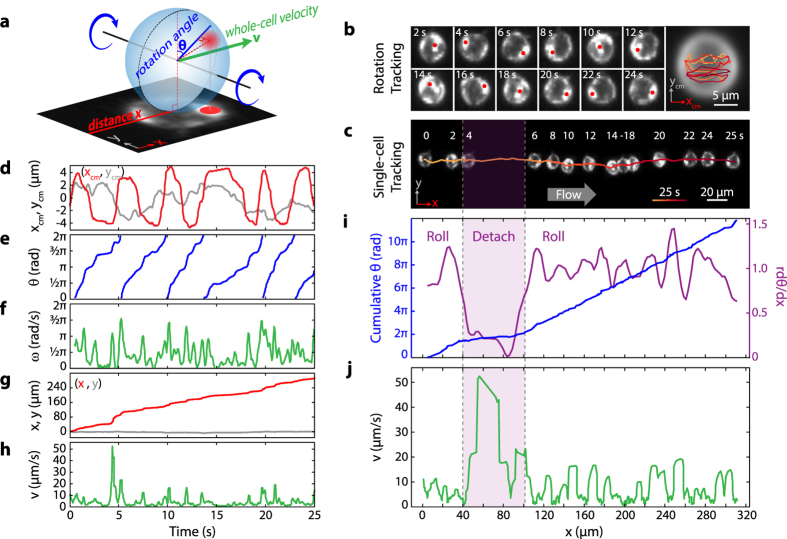
Single cell translation and rotation tracking. The rolling cell is under a constant shear stress of 0.12 Pa. (**a**) Schematic of a rolling cell with bright spot (red) with its projected 2D image on the *x*-*y* plane. *x* is defined along the flow direction. (**b**) Rotation tracking of a single cell. One (or more) spots from isolated cell images at different time points are identified (red circles) and analyzed to form a single trajectory in the frame of reference of the cell *x*_*cm*_*-y*_*cm*_ (right panel). Time is represented by the orange-to-red color gradient. (**c**) Whole-cell tracking with snapshots of the same cell at 2-s intervals in the lab reference frame *x*-*y*. Time is represented by the same color gradient as in (**b**). (**d**) Trajectories of the *x* and *y* positions of the single spot from (**b**) in the cell reference frame *x*_*cm*_-*y*_*cm*_. (**e**) Reconstructed rotation angle *θ* of the cell. (**f**) Angular velocity *ω* as calculated from *θ*. (**g**) Trajectories of the *x* and *y* positions of the cell centroid from (**c**). (**h**) Whole-cell rolling velocity *v* as a function of time calculated from (**g**). (**i**) Cumulative angle *θ*, the normalized slope *rdθ/dx* (values around 1 indicate the cell is maintaining traction, values ≤0.5 indicate transient detachment) as functions of rolling distance *x* corresponding to the images in (**c**). (**j**) Cell velocity *v* as a function of rolling distance. The purple band from 25–63 s indicates the period where the cell transiently detached from the surface and freely floated in the flow before it was recaptured and started rolling again.

Using custom image analysis software, we separated cell rolling motion into rotational (Fig. 2a,b–f) and translational (Fig. 2c,g,h) components (Supp. Figure S2). The additional information contained in rotational motion provides important advantages over translational motion alone. Cell rolling frequently involves transient detachment and reattachment, where the cell loses contact with the surface and floats freely for short distances (Fig. 2c,i,j at *x* = 40–100 μm). For rolling cells maintaining traction with the surface, the angular velocity and translational velocity are related by *v* = *rω*, where *r* is the cell radius. This can be seen from the linear relation between the cumulative rotation angle *θ* and displacement *x* (Fig. 2i, 100–300 s range). Freely floating cells also rotate due to the shear flow near the surface, but this rotation happens at a much lower degree of rotation per distance travelled compared to rolling cells on a surface. As a result, *v* ≫ *rω* for transient cell detachment, which can be easily distinguished from the derivative *dθ/dx* = *ω/v* (Fig. 2i). Furthermore, knowing the orientation of the cell allows us to track directly and reliably the substrate contact point on the cell surface even when the cell temporarily loses traction with the surface, in which case translation and rotation are not simply related by cell radius.

Within the camera field of view, cell rolling trajectories are approximately straight with minimal side-to-side deviation, indicating that a narrow band of cell surface along the vertical circumference makes contact with the substrate (Fig. 2a). By tracking the rotational and translational motion of a single cell, we can map its adhesion properties around this contact circumference. We used a dwell time per unit angle defined by *τ(θ*) = 1/*ω(θ*) as a proxy for the adhesive strength at a particular contact point and time. This definition borrows from the concept of single molecule bond strength^26^, which is usually measured as bond lifetime at a given force. Bonds with longer lifetimes are stronger than bonds with shorter lifetimes. In the case of cell rolling, although we are not measuring single-molecule bond life-time, we can think of longer lasting cell-surface contacts as more adhesive than shorter duration contacts. *τ* is the duration of a cell-surface contact per unit angle, i.e. the inverse of the angular velocity *ω*. Hence, *τ(θ*) defines the overall behaviour of cell adhesion at the contact patch due to a sum of effects such as adhesion bond lifetime, receptor density and tether extension.

**Figure 3:**
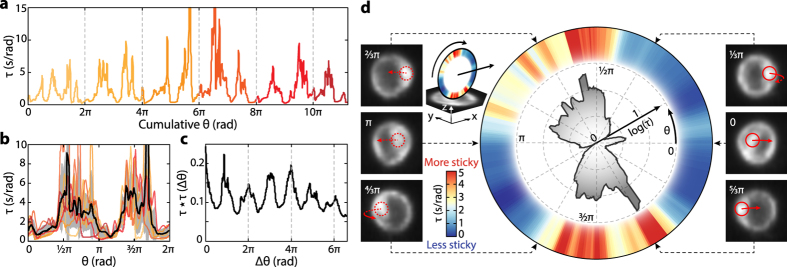
Single cell surface adhesion map from rotation tracking of the same cell as Fig. 2. (**a**) Dwell time *τ* as a function of cumulative angle *θ* of a single cell. Each color corresponds to one 2π rotation period. (**b**) Dwell time *τ* from (**a**) plotted vs. overlapping 2π periods. Same color code as in (**a**). The black solid line is the average dwell time over the 2π period, and the gray shaded area is the standard deviation. (**c**) Normalized autocorrelation of *τ(θ*) in (**a**). (**d**) Polar representation of the average dwell time in (**b**). *τ(θ*) is represented in two ways: as a line plot on a log scale (black line & shaded gray area), and as a linear color map, with red and blue showing stronger and weaker adhesion, respectively (lower inset). Upper inset illustrates the rolling direction of the polar plot. Six cell images display the average cell image at the particular rotation angle denoted by the dashed black arrows. The bright spot position for this cell is marked by the red circles (solid when the spot is located on the top cell surface, dotted when on the bottom surface) and the rolling direction by the red arrow. There is no correlation between the spot position and adhesion strength. See Supp. Figure S5 for a gallery of cell adhesion map disks.

At first glance, the dwell time as a function of cumulative angle may not show clear patterns (Fig. 3a). However, since the cell rolls repeatedly along the same contact circumference, it is possible to stack the dwell time within one 2π period (Fig. 3b). A pattern emerges from this exercise; for this particular cell, the dwell time in the angle range of [0.4π, π] and [1.3π, 1.7π] is significantly longer than in the range of [π, 1.2π] and [−0.2π, 0.4π]. The average dwell time (Fig. 3b, black line) spans a range over an order of magnitude over the cell circumference. Although the dwell time on small angular scales (≪π/2) shows uncorrelated fluctuations (Fig. 3b, black line), likely the result of stochasticity in adhesion events, it shows positive correlation on larger angular scales (~π/2). The autocorrelation function of *τ* over cumulative *θ* shows clear peaks at integer multiples of 2π, indicating significant dwell time correlation over each full rotation cycle (Fig. 3c). The additional peaks in the autocorrelation for this particular cell are a coincidental result of its two large adhesion patches nearly π phase apart. Other cells show a singular strong adhesion patch or multiple patches that are distributed randomly around the cell (Supp. Figure S5). Figure 3c shows a polar representation of the average dwell time, color-mapped onto the contact circumference to provide a direct visualization of the adhesion properties around this cell.

**Figure 4:**
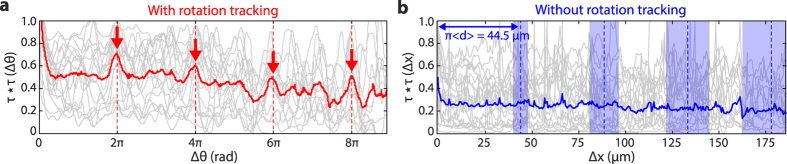
Asymmetric adhesion across HL-60 population. (**a**) Normalized autocorrelations of the dwell time *τ(θ*) measured as a function of rotation angle *θ* for many individual cells (*N* = 30; gray) and their average (red). The average autocorrelation displays peaks at 2*n*π (*n* = 1, 2, 3, 4; red dotted lines and arrows), indicating periodicity in adhesion. (**b**) Normalized autocorrelations of the dwell time *τ(x*) measured as a function of position *x* for the same population of cells (gray) and their average (blue). Without rotation tracking, the autocorrelation fails to display peaks at distances corresponding to multiples of the average circumference *π*<*d*>, where <*d*> is the average cell diameter (blue dotted lines; blue shades indicate the standard deviation due to cell size variations. Both the average and standard deviation are obtained from the average cell diameter, as shown in Fig. 5).

We analyzed *N* = 30 cell trajectories for which we could track individual spots for a minimum of 4 rotation cycles. The autocorrelation functions of *τ(θ*) for these cells all exhibit a 2π period (Fig. 4a; gray lines). Averaging the autocorrelation functions over all cells (Fig. 4a; red line), only peaks at integer multiples of 2π sum up constructively. The peaks at 2*n*π indicate that the adhesion patterns repeating each rolling cycle is a property shared by all HL-60 cells. We determined from the width of the autocorrelation peaks that the average characteristic size of the adhesion patches is ~1/4 of the rolling circumference, but can vary from 1/12 to 5/12 as seen from Supp. Figure S5. In a control where the same 30 cell trajectories were analyzed using translational tracking only, no periodicity emerged in the autocorrelation function of the dwell time (Fig. 4b). Transient cell detachments and cell-size variations wash out the repeating adhesion pattern, underscoring the advantages of rotation tracking.

### Adhesion Footprint Assay

The periodic rolling behavior of HL-60 cells strongly indicates systematically asymmetrical adhesion properties along the rolling contact circumference. To confirm that the observed pattern results from the adhesion properties of the cell, we set out to record the molecular adhesion footprint of the rolling cell. Rolling adhesion is highly dynamic and single adhesion bonds are believed to form and dissociate on sub-second time scales^12^,^17^. In addition, only a small fraction of available receptors on the substrate is used in the adhesion^17^. Traditional elastomer-based molecular force sensors^27^,^28^,^29^ are not suitable to visualize rolling adhesion as very high signal-to-noise ratios are required to detect these rare events. Thus, we developed a DNA-sensor-based fluorescence assay to record the molecular footprint. The assay is derived from the “Tension Gauge Tether” approach recently reported^20^,^30^.

**Figure 5:**
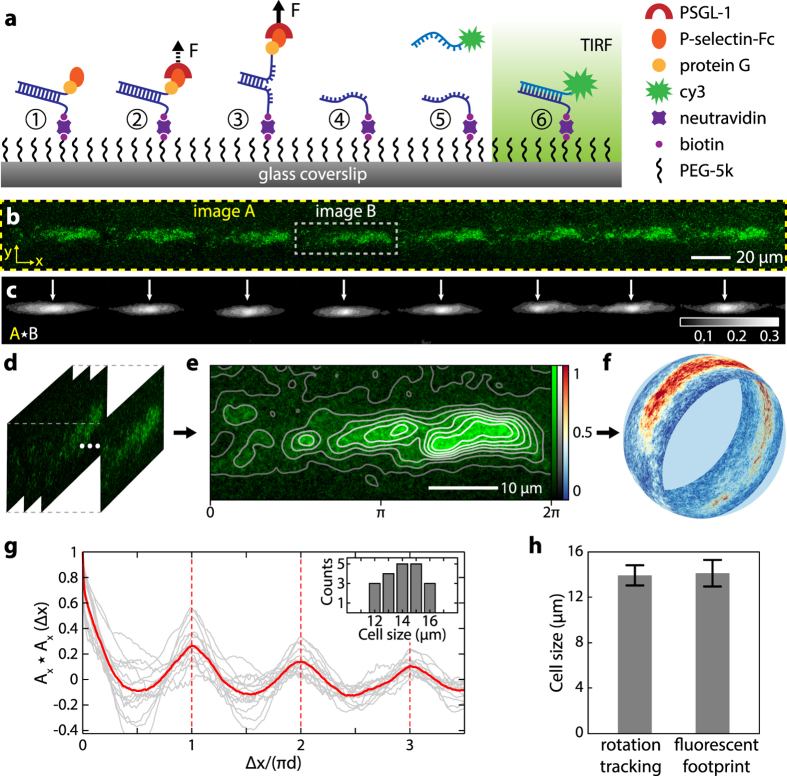
Adhesion asymmetry in rolling cells revealed by molecular adhesion footprinting. (**a**) Schematic of the molecular adhesion footprint assay. A surface is coated with P-selectin-functionalised DNA molecular sensors (Step 1). Adhesion of PSGL-1 and application of force (Step 2) unzips the DNA sensor (Step 3), leaving single-stranded (ss)DNA at the adhesion site on the surface (Step 4). Complementary Cy3-labeled DNA flowed into the chamber (Step 5) hybridizes with unzipped sensors (Step 6), marking their location for fluorescence imaging. (**b**) Representative molecular adhesion footprint of a rolling cell (image A) by Cy3-fluorescence imaging of the surface under TIRF illumination. Cell rolling was performed under a calculated shear stress of 0.12 Pa. (**c**) Normalized 2D cross-correlation of image A with its subset image (image B). Color map displays the numerical value of the cross-correlation. Arrows indicate the horizontal positions of the local cross-correlation maxima and reveal the periodicity in the adhesion footprint. (**d**) Images cropped from image A, with width corresponding to the period from the cross-correlation in (**c**) and centered about the positions of the local cross-correlation maxima. (**e**) Average of the fluorescence images from (**d**) over a single-period (0–2π) and a linear contours plot of fluorescence intensity from background (value = 0) to maximum (value = 1). (**f**) Map of the 2D molecular adhesion footprint from (**e**) onto the surface of a sphere, representing the contact circumference of the cell. Color scales linearly with fluorescence intensity in (**e**). (**g**) Normalized autocorrelations of the fluorescence footprint along the *x* axis for individual cell tracks (*N* = 20; gray), and their average (red). The *x* axis is normalized by the individual cell circumference (π*d*). Inset shows the histogram of cell diameter. (**h**) Average cell diameter from video rotation tracking (14.1 ± 1.0 μm) and fluorescent footprint (14.2 ± 1.2 μm) agree with each other.

In this assay, 18-base pair DNA duplex with biotin- and P-selectin-modified complementary strands are immobilized on PEG-passivated glass coverslips (Fig. 5a; step 1). HL-60 cells roll on these modified surfaces via selectin-specific interactions. During cell rolling, adhesion of PSGL-1 on the cell surface to P-selectin applies an unzipping force to the DNA duplex, and if the force applied exceeds the DNA rupture force the DNA strands are separated, leaving only the bottom strand attached to the surface (step 2–4). Dye-labeled oligonucleotides complementary to the lone bottom strand are then flowed in to hybridize to the ruptured DNA strand to form a new duplex (step 5). This assay thus reports the locations of single ruptured DNA duplexes through detection of fluorescence emission (step 6), where molecular adhesion between the rolling cell and the surface takes place. The assay has extremely high sensitivity as only ruptured DNAs produce signal whereas unperturbed DNA duplexes emit no light with only rare strand exchange processes happening during the brief annealing process (step 5). Since the TGTs respond to force approximately digitally (at ~12 pN as previously estimated^20^,^30^) and the fluorescence signal level is directly proportional to the number of ruptured DNA, this assay allows us to quantify the surface density of adhesion events above a pre-defined DNA rupture force.

Consistent with the adhesion map derived from rotation tracking, fluorescent adhesion footprints of HL-60 cells also show repeating patchy patterns (Fig. 5b, and Supp. Figure S6). The 2D cross-correlation between the image of a single repeating unit and the image of the entire footprint shows the repeating pattern more conspicuously (Fig. 5c). The distance between peaks in the 2D cross-correlation map is consistent with the circumference of the cell, measured separately from rotation tracking. Isolating each repeated pattern (Fig. 5d), we determined an average adhesion map (Fig. 5e), representing the mean functional adhesion properties along this particular contact circumference on the cell surface (Fig. 5f). The pattern is highly asymmetrical with a portion of it being strongly adherent and others weakly adherent. Analyzing *N* = 63 tracks, most (*N* = 51, or 81%) adhesion footprints exhibited periodicity. Among these, *N* = 20 tracks that did not cross other tracks were isolated for autocorrelation analysis (Fig. 5g). The periodicity matched well the mean cell circumference obtained from whole cell tracking (Fig. 5h). In control experiments with PSGL-1 modified rolling beads, the adhesion footprints were uniform and exhibited no clear periodicity (Supp. Figure S4a–d). The fluorescent footprint also allows us to assess cell deformation from direct measurement of the cell-surface contact width (5.7 ± 1.3 μm, Supp. Figure S6b,c). This correspond to a cell contact area of 26 ± 12 μm^2^ assuming a circular contact area, or 4 ± 2% of the total cell surface area. The small contact area also suggests that the cells are not significantly deformed (4% height change) under flow (Supp. Figure S6b). Of the cells that did not exhibit clear periodicity, it is possible that these cells lack the adhesion asymmetry or that the rotation plane shifted during rolling, such that the adhesion pattern did not repeat.

Rolling adhesion models^31^,^32^,^33^ have assumed a uniformly stochastic distribution of microvilli and adhesion molecules based on fluorescence imaging of PSGL-1 antibodies on leukocytes^21^. Consequently, variations in rolling velocity were explained by the stochastic nature of adhesion bond rupture alone. Our results challenge this view and show that such variation has a large contribution from the systematic adhesion asymmetry at the behavioral and the molecular levels. Such adhesion asymmetry should be taken into consideration when creating quantitative models of rolling cells.

## Discussion

What mechanisms could give rise to this asymmetry ? Spatial variation in the distribution of adhesion molecules is highly unlikely to be due purely to statistical fluctuations, given the large surface contact area of HL-60 and the large number of adhesion molecules per cell (~17 PSGL-1 per microvilli and ~730 microvilli per cell^33^). Control experiments using 6-μm polystyrene beads coated with PSGL-1 show extremely uniform rolling velocity on P-selectin-coated surfaces, as compared to HL-60 cells. The majority of beads (90%) exhibited no signs of periodic adhesion patterns from tracking (Supp. Figure S3a,b) or from fluorescence adhesion footprinting (Supp. Figure S4e). Assuming a random distribution of PSGL-1 on bead and cell surfaces, one would expect the significantly smaller surface contact area of beads (~2 μm^2^ vs ~80 μm^2^) to have led to a significantly greater variation in rolling velocity in beads than in cells. This is contrary to our observation, pointing to a systematic spatial inhomogeneity rather than stochastic variation as the source of the strong adhesion asymmetry observed for HL-60s. The 10% of beads that showed periodic adhesion patterns are likely the result of bead aggregation during antibody incubation, resulting in uneven surface coating. To confirm that a random spatial distribution of microvilli on the cell surface is not responsible for the observed large asymmetry, we carried out simulations of rolling spheres with randomly distributed microvilli on their surface, using experimentally determined parameters^33^,^34^,^35^ (Supp. Methods). We determined the number of microvilli that makes contact with the surface during cell rolling assuming all microvilli consistently form tethers every rotation cycle (Supp. Figure S9). The simulation result shows that although repeating patterns emerge as expected over perfectly replicated rotation cycles (Supp. Figure S9f), neither the number of tethers (Supp. Figure S9b–d,g,h), nor their expected adhesion footprint (Supp. Figure S9e,f) shows the strong intensity and spatial variation observed experimentally (Figs 3 and 5b). Therefore, a density variation due to a random spatial distribution alone is statistically unable to produce significant density variations over large patches.

**Figure 6:**
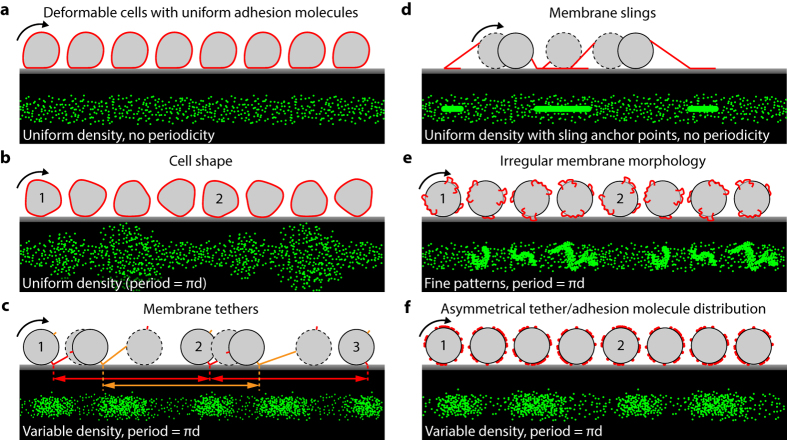
Cartoon illustrations of fluorescent footprint patterns due to isolated cell shape, receptor, and membrane morphology perturbations. (**a**) A cell with uniform receptor distribution deforming due shear flow compression alone would produce uniform tracks without periodic patterns, much like those in a bead rolling assay (Supp. Figure S4A). (**b**) An undeformable cell with irregular cell shape but uniform surface receptor distribution would leave a periodic footprint that is uniform in intensity but variable in width. (**c**) Short elastic membrane tether formation does not alter periodicity of rolling as long as the cell maintains traction (the figure exaggerates the length of each tether to illustrate the concept; in reality, the tethers are only a few hundred nm in length). The tip of each tether (red and orange) forms adhesive contacts after each rotation cycle of the cell, regardless of the elastic tether history. (**d**) Recently discovered membrane ‘slings’ that are thought to stabilise cell rolling under high shear conditions^42^ would only produce contact adhesion points at discrete intervals, which is unlikely to produce periodicity, if any, that matches cell circumference. (**e**) A small fraction of cells that exhibit irregular membrane morphology such as small ruffles would introduce periodicity in the fluorescent footprint as repeating fine patterns that match the shape of the membrane irregularities. (**f**) Asymmetrically distributed receptors would produce periodic patterns with variable fluorescent intensity.

We considered several scenarios that could affect cell rolling behavior and adhesion periodicity (Fig. 6). Cells are known to deform upon contact with the surface due to shear flow (Fig. 6a). It is increasingly recognized that deformation is important for cells to sustain rolling under high flow rates by increasing their surface contact area and decreasing shear and drag force^36^,^37^,^38^. Although individual HL-60 cells are not perfectly spherical in shape, cell deformation under flow is expected to decrease the variation in contact area, making variations in cell shape unlikely as a dominant factor for adhesion asymmetry (Fig. 6b). Membrane protrusions or microvilli tether the cell body to the adhesion molecules. Experiments have shown that microvilli are extendable under tension and serve as force buffers that control the total amount of force transmitted to the adhesion molecules at its tip^39^,^40^,^41^. Their length, number and attachment geometries also influence the rolling behavior^24^,^40^,^42^. Most microvilli are elastic with lengths on the order of hundreds of nm. When cells maintain traction with the surface, these membrane tethers reform after each rotation cycle (Fig. 6c). Hence, normal membrane tethers that are a key part of the rolling adhesion process do not interfere with the periodicity observed here. Recent rolling cell studies have reported highly extended microvilli or “membrane slings” that span several cell diameters but exist in low copy numbers^42^. During cell rolling, membrane slings catapult ahead of a cell and lay adhesive tracks that are believed to contribute to stable rolling under high shear stress^42^. However, these rare membrane slings do not contribute to the adhesion asymmetry. Both rotation tracking and adhesion footprint point to a precise *πd* periodicity of adhesion, where *d* is the cell diameter, indicating that the asymmetrical adhesion properties reside close to the cell surface (adhesion molecules and short typical microvilli tethers). Membrane slings beyond the cell diameter would generate adhesion periodicity other than *πd*, because the distance between consecutive anchor points would incorporate both the cell circumference and tether lengths (Fig. 6d). In addition, the slings would leave distinct lines in adhesion footprint assay (Fig. 6d), which we did not observe under our experimental conditions. Although microvilli are the major type of membrane protrusion on undifferentiated HL-60 cells, other membrane structures near the cell surface such as short membrane ruffles that occasionally occur in undifferentiated HL-60^43^ are also a potential candidate for the observed adhesion asymmetry (Fig. 6e). However, their occurrence is much rarer than the population of cells that possess the rolling asymmetry^43^. Therefore, for the majority of cells that show adhesion asymmetry, it most likely reflects differences in local surface protein density (Fig. 6f).

It has been suggested that the oscillatory motion of rolling adhesion involving L-selectin serves as a braking mechanism that stabilizes the rolling process^44^. A similar mechanism may be utilized by cells to stabilize rolling on P-selectin. In addition, rolling adhesion contains an important mechanical signaling step toward subsequent integrin activation and firm adhesion^2^. We speculate that asymmetrical adhesion properties may also contribute to mechanical signaling events. Asymmetrical adhesion allows certain adhesion molecules to experience stronger forces than others, potentially triggering their activation. It has been shown that cells react differently to oscillatory mechanical forces compared to constant forces^45^,^46^,^47^,^48^. It is thus conceivable that leukocyte rolling with asymmetrical adhesion properties could provide a means to achieve periodic force perturbations for a more effective signal response. More studies will be necessary to determine if such a mechanism is plausible.

Simultaneous orientational and positional tracking provide significantly more information than either one alone. By labelling a single viral particle with quantum dots, Kukura *et al*. observed both sliding and tumbling motions of the viral particle on plasma membrane as a result of protein binding fluctuation^49^. Using fluorescent Janus particles, Suo *et al*. were able to quantify the force and torque on a rolling microsphere under shear flow, which differed by close to 25% from classic fluidic dynamic models that have been used for over 50 years^50^. The label-free cell rotation tracking and adhesion mapping platform we developed for studying cell rolling adhesion enabled us to create functional maps of cell surface adhesion properties. This allowed us to correlate cell motion directly with precisely mapped cell surface adhesion properties. Traditionally, immuno-fluorescence labelling of cell surface receptors can provide the locations of target receptors on the surface. This, however, does not necessarily correlate with cell surface adhesion at the functional level due to cell shape and contact geometry. In addition, a disadvantage of immuno-fluorescence labelling is that the antibodies usually block the active sites or alter the target protein’s native function. Thus it is not suitable for simultaneous imaging and cell function studies. Although AFM-based adhesion force map measurements do not require target protein labelling and provide high spatial resolution^51^, their speed and the region of cell surface they can sample are inherently limited. Rolling adhesion being highly dynamic, cells simply roll too fast to be imaged by AFM. While fixing cells in place is possible, the AFM tip geometry limits the region it can probe on the upper cell surface. Our method provides notable advantages over the above methods in that it maps adhesion properties on the cell surface without the need to label or modify the cell and also in an intrinsically high-throughput manner.

Although the present approach maps adhesion strength along the rolling circumference of the cell, we believe it can be extended to probing its entire surface. In addition, DNA sensors with different rupture forces can be used to reveal the range of adhesion forces at play in rolling adhesion. This method can be easily extended to study the surface adhesion properties of other cell types, including different types of white blood cells and circulating cancer cells. For cells that do not contain granules, the cell surface in principle can be tagged sparsely by functionalized gold nanoparticles, which strongly scatter light and are visible under darkfield microscopy. Quantitative understanding of the adhesion behavior of rolling cells is important not only for fundamental cell biology, but also for practical applications including cancer diagnostics, screening and treatment^9^,^10^,^11^. The approaches presented here represent a first step toward a comprehensive label-free cell surface adhesion mapping and characterization technique.

## Methods

### Cell culture

HL-60 human leukemia cells (ATCC, CCL-240) were cultured according to ATCC recommended protocols. Briefly, Iscove’s Modified Dulbecco’s Medium (IMDM, ATCC 30-2005) with 20% fetal bovine serum (ATCC, 30-2020) was used as the growth medium. Growth medium was renewed every 3 days by 1:10 dilution of fresh medium in a cell culture flask with vent cap (Corning, 430639) to ensure that the maximal cell density did not exceed 10^6^ cells/mL. Cells for experiments were prepared by washing in Ca^2+^- and Mg^2+^- free HBSS via centrifugation and were immediately used in experiments.

### Surface preparation

Glass coverslips were PEGylated as described previously^52^. Briefly, glass coverslips were cleaned by 15 min sonication in acetone followed by 1-hour sonication in 3 M KOH. Following amino-silanization (UCT Inc., A0700-KG), a monolayer of mPEG (Laysan Bio, MPEG-SVA-5000) was covalently linked to the coverslip surface to form a uniform passivation layer that prevents non-specific interactions between cell and surface. Biotinylated PEG (Laysan Bio, Biotin-PEG-SVA-5000) was added to mPEG at a 1:100 molar ratio to provide anchor points for subsequent protein immobilization. Protein immobilization was done *in situ* by first saturating surface biotins with 0.5 mg/mL neutravidin, then incubating a premixed 250 nM solution of biotinylated Protein G (Thermo Scientific Pierce, 29988) with recombinant human P-Selectin Fc chimera (R&D Systems Inc., 137-PS-050) at a 1:1 molar ratio. All incubations were performed at room temperature for 30 min; each incubation was followed by washing with PBS (pH 7.4). The passivation protocol was originally developed to block non-specific protein adhesion on surfaces for single-molecule fluorescence studies^52^. Such thorough passivation ensures specific interaction between cells and surface immobilized proteins. Control experiments show that stable rolling is only achieved when all tethering proteins are present (Supp. Figure S8).

### Fluidic device setup

A custom-made fluidic device was used for all cell rolling experiments with channel dimensions of 80 μm × 2 mm × 50 mm. First, a laser engraver (VersaLaser VLS 2.30) was used to cut out well-defined channels in double-sided tape (3 M Scotch 665). The tape was then sandwiched between two glass coverslips to make the channel. Holes were cut by laser engraver on one of the coverslip for inlets and outlets to the flow channel. Polyethylene tubings (BD Intramedic, 427406) were coupled to the inlets and outlets on the coverslip using custom-made anodized aluminum frame. Cells were introduced to the channel by flow using a syringe pump (Harvard Apparatus). Various flow rate from 100–5000 μL/hr were used in the experiments. This corresponds to physiological range of surface shear stress *τ*_*w*_ of: 0.01–0.51 Pa, as calculated by:


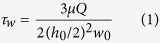


where *μ* is the viscosity of water; *Q* is the flow rate; *h*_*0*_ and *w*_*0*_ are the height and width of the flow chamber cross-section^53^. HBSS with 2 mM CaCl_2_ was used as flow buffer in all experiments.

### Bead preparation

Protein G-coated polystyrene beads (OD 6.6 ± 0.4 μm) were purchased from Spherotech (PGP-60-5). 10 μL of 0.5% w/v beads in stock solution were washed twice in PBS by centrifugation and removing supernatant. The beads were then incubated with 50 μL of 100 μg/mL PSGL-1-Fc Chimera (R&D systems, 3345-PS-050) for 2 hours on a rotator in room temperature before experiment. All beads were washed in PBS (pH 7.4) prior to experiments.

### Dark-field Microscopy

Cells in the flow chamber were imaged by dark-field using a Nikon TE-2000 microscope. A 10x objective with a 2.5x tube lens was used to achieve a combined 25x magnification. Images were recorded using an Andor iXon3-897 camera at 30 fps. Unless otherwise noted, all tracking was done in 25x.

### Cell tracking

Custom image analysis programs were written in Matlab to track both the translational and rotational motions of individual cells. Cells were identified in each frame by a binary threshold and connected component algorithms (built-in Matlab functions). The translational motion (Fig. 2c) was determined by calculating and connecting the centroids of each isolated binary cell image. The resulting cell tracks provided similar information as conventional cell rolling studies^19^, including the positions (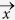) and instantaneous velocity (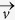) (Fig. 2g,h,j). In order to isolate the rotational motion, a movie of an individual cell in the reference frame of its centroid was generated (Fig. 2b and Supp. Figure S3a,b). The positions of the spots inside individual cells projected in 2D (Fig. 2b,d) was determined by tracking feature points generated by a Maximally Stable Extremal Regions (MSER, built-in Matlab blob feature detection function) analysis of each isolated cell frame (Supp. Figure S3c). Trajectories of feature points from a single spot were tracked (Supp. Figure S3d). The maximal distance in these trajectories yield the actual radial distance *R* between the spot and the cell center. The relative rotation angle *θ* can then be calculated as:


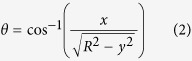


A single trajectory of relative *θ* is constructed by averaging over the trajectories of each feature point (Supp. Figure S3g). In this case, the relative angle has a range of 0 − π due to the degeneracy of the cos^−1^ function. This degeneracy can be removed by flipping alternating 0 − π intervals about π by the following operation: *θ*′ = 2π − *θ* (Fig. 2e). The cumulative *θ* can then be calculated by 2π offsets every time the relative angle reaches 2π (Fig. 2h and Supp. Figure S2h). The slope of cumulative *θ* as a function of *x* gives a precise measurement of the cell circumference unbiased from cell deformation and imaging condition (Supp. Figure S2h). We estimated the tracking precision to be 0.5 μm, which translates to 2° of angular uncertainty.

### DNA constructs for adhesion footprint assay

The adhesion footprint construct (Fig. 5a(1)) was derived from the recently developed DNA-based tension gauge tether (TGT) method^20^,^30^. Briefly, P-Selectin Fc chimera was attached to the top strand of the DNA TGT via a covalently conjugated protein G (ProtG). The bottom strand was biotinylated and annealed to the top strand via complementary base-paring. The bottom strand of the TGT was attached to the surface via biotin-neutravidin interactions. The probe strand (Fig. 5a, step 5) was conjugated to a Cy3 dye. The following oligonucleotides with modifications were purchased from Integrated DNA Technologies (IDT):

Top: 5′-ACACACACCCTTTTTTTT /3ThioMC3-D/-3′

Bottom: 5′-/5BiosG/TTATTAAAAAAAAGGGTGTGTGT-3′

Probe: 5′-ACACACACCCTTTT/3AmMO/-3′

Protein G (Abcam ab49807) was conjugated to the top strand via sulfo-SMCC (Thermo Scientific, 22122) crosslinking agent, following protocols recommended from the manufacturer. The concentration of the conjugation product (Top-ProtG) was determined by UV-vis to be 16 μM. TGT-ProtG was made by mixing Top-ProtG and bottom strand at 1.2:1 molar ratio to ensure excess Top-ProtG. P-selectin-Fc was incubated with TGT-ProtG at a 1.2:1 molar ratio to ensure excess P-selectin-Fc. Incubations were performed at room temperature for 30 min. Buffer common to all dilutions and incubations was PBS (pH 7.4). Cy3 (GE healthcare life science, PA23001) was conjugated to the probe strand by NHS-ester conjugation chemistry following manufacturer protocols.

### Adhesion footprint assay

TGT-ProtG was immobilized on PEGylated glass coverslips *in situ* by first incubating the flow chamber in 0.5 mg/mL neutravidin for 30 min, then incubating it in 100 nM TGT-ProtG:P-selectin-Fc. The surface density of the protein is estimated to be ~400/μm^2^ (based on the surface density of biotinylated PEG). All incubations were performed at room temperature for 30 min; each was followed by washing with PBS (pH 7.4). Cells (or beads) were introduced into the flow channel in IMDM and allowed to roll on TGT-ProtG:P-selectin-Fc the functionalised surface for 1–2 min at shear stresses between 0.05–0.20 Pa. The channel was then flushed with IMDM at a 5 Pa shear rate to detach all cells (or beads). 100 nM Cy3-labeled probe oligonucleotide was then introduced into the channel in T50 buffer (10 mM Tris and 50 mM NaCl) and allowed to incubate for 10 s before flushing out with excess T50 buffer.

### TIRF microscopy & fluorescence image processing

Images of fluorescent footprints were acquired with a Nikon CFI Apo TIRF 100x objective on a Nikon Ti-E inverted microscope. TIRF illumination was achieved with a 514-nm Argon laser. Images were recorded with an Andor iXon3-897 EMCCD camera. Overlapping image tiles were collected with a motorized microscope stage using the built-in features in the Nikon microscope control software (NIS-element AR).

An uneven illumination intensity profile caused fluorescence intensity changes across each image (Supp. Figure S7a). Each image was corrected using the following formula: *I*_*corrected*_ = (*I*_0_ − *I*_*bg*_)/*I*_*illumination*_ where *I*_*corrected*_ is the corrected image, *I*_0_ is the original image, *I*_*bg*_ is the background noise image without illumination, and *I*_*illumination*_ is the normalized illumination profile with a maximum of 1. The illumination profile *I*_*illumination*_ was determined by the normalized average of hundreds of images in each dataset <*I*_0_ − *I*_*bg*_> and scaled by its maximum (Supp. Figure S7b). Stitching between adjacent images was performed by matching overlapping features manually and finding the pixel offsets in both dimensions (Supp. Figure S7f). The images were then cropped and joined without averaging. Controls were performed to ensure that such background correction and stitching did not introduce artificial periodicity the size of image repeats (Supp. Figure S7d,e). The resolution of the fluorescence footprint assay is essentially limited by the optical resolution of ~250 nm.

## Additional Information

**How to cite this article**: Li, I. T. S. *et al*. Mapping cell surface adhesion by rotation tracking and adhesion footprinting. *Sci. Rep.* **7**, 44502; doi: 10.1038/srep44502 (2017).

**Publisher's note:** Springer Nature remains neutral with regard to jurisdictional claims in published maps and institutional affiliations.

## Supplementary Material

Supplementary Information
