# Supplementary material for: Mapping cell surface adhesion by rotation tracking and adhesion footprinting
Source: Sci Rep. 2017 Mar 14;7:44502. doi: 10.1038/srep44502 (PMC5349612; doi:10.1038/srep44502)

**Supplementary Information**

**Mapping cell surface adhesion by rotation tracking and adhesion footprinting**

Isaac T.S. Li ^a,1^, Taekjip Ha ^a,b^, Yann R. Chemla ^a,*^

^a^ Department of Physics and Center for Physics of Living Cells, University of Illinois at Urbana-Champaign, 1110 W Green St., Urbana, IL, 61801, USA; ^b^ Howard Hughes Medical Institute, Department of Biophysics and Biophysical Chemistry, Department of Biophysics and Department of Biomedical Engineering, Johns Hopkins University, 725 N. Wolfe Street, Baltimore, MD, 21205, USA

^1^ Current address: Department of Chemistry, University of British Columbia, 3247 University Way, Kelowna, BC, V1V1V7, Canada

^*^ Corresponding Author: Yann R. Chemla; 1110 W Green St., Urbana, IL, 61801, USA; Phone: 217.333.6501; Email: [ychemla@illinois.edu](mailto:ychemla@illinois.edu)

**Supplementary Methods**

**Simulations of adhesion footprints**

We simulated rolling cells with a random but uniform spatial distribution of microvilli. A microvilli density of 4 μm^-2^ was used based on previous studies ^1-3^ to generate an isotropic distribution on the surface of a sphere (Supp. Figure S9a). Microvilli were given the following coordinates:

where *r*_1_ and *r*_2_ are random variables. The diameter of the sphere was 14.1 μm according to the measured mean cell size (Figure 5h). Surface-contacting microvilli were defined as those within a contact circle 5.7 μm in diameter according to the measured mean footprint width (Supp. Figure S6b). The number of surface-contacting microvilli was tracked as a function of the rotation angle of the cell (Supp. Figure S9c,d). The microvilli footprint (Supp. Figure S9e) was generated by tracking the contact coordinates of individual microvilli as the cell rolled.

**Supplementary Figure Legends**

**Supplementary Figure S1.** Intracellular markers are stationary. (a) Dark-field images of a cell immobilized on a P-selectin surface under no flow (60x magnification). The bright spots inside the cell remain static over the course of a minute. Scale bar is 5 µm. (b) Tracking of individual internal spots over a period of 90 s inside one immobilized cell. (c) Mean squared displacement (MSD) vs. time analysis showing normal diffusion with a diffusion coefficient of 0.023 μm^2^/s. The shaded area indicates typical observation duration for cell rolling across the field of view. The mean displacement over a 20s period is ~0.67 μm, which is ~5% of the cell diameter, while cells on average complete 5 rotation cycles. Variation due to spot diffusion is a much smaller source of error compared to other processes such as tracking and motion blur. Hence the mobility of spots within the cell can be ignored within the time frame of our experiments.

**Supplementary Figure S2.** Rotation tracking procedure. (a) Snapshots of a single cell as it rolls on the surface. Images of the cell following its center-of-mass reference frame were isolated by cropping (white boxes) the original movie. Isolated images of a single rolling cell (b) were then processed by the MSER feature detection method (built-in function in Matlab) to identify key feature points (red dots) (c). (d) Trajectories of a single moving spot. (e) The *x* and *y* components of the trajectories as a function of frame number. (f) Relative angle of a feature point calculated based on the location of the point (e) and its maximal distance to the cell center. (g) Average trajectory of (f) as a function of distance *x*. (h) Cumulative angle as a function of *x* reconstructed from (g).

**Supplementary Figure S3**. Rolling beads coated with PSGL-1 do not exhibit adhesion asymmetry. (a) Normalized autocorrelations of dwell time *τ*(*x*) measured as a function of position *x* for individual rolling beads (*N* = 35; gray) and their average (blue). The average autocorrelation shows no adhesion periodicity at multiples of the expected bead circumference (blue dotted lines; blue shaded area indicates the standard deviation from bead size variation). Inset shows the size distribution of the beads. (b) Histogram comparing the fraction of cells and beads exhibiting periodic adhesion patterns.

**Supplementary Figure S4**. Fluorescence footprinting for 6-μm diameter PSGL-1 coated beads. (a) Representative molecular adhesion footprint (image a) of the surface by Cy3-fluorescence imaging under TIRF illumination. (b) Normalized 2D cross-correlation of image A with its subset image (image B) shows no detectable patterns, in contrast to the periodic repeats in Figure 5B. (c) Intensity projection of image A in the *x* direction. (d) Normalized autocorrelation of the intensity in (c) showing no clear peaks at distances corresponding to multiples of the expected circumference (black arrows; dotted gray lines represented the range in expected periodicities based on the known variance in bead diameter from Supp. Figure S3). (e) Representative fluorescence image showing multiple bead tracks in the same field of view. The intensities are uniform along all tracks, displaying no periodic patterns.

**Supplementary Figure S5.** Gallery of adhesion maps of 20 different cells showing averaged adhesion pattern over the cell circumference. Each map was generated following the same workflow as shown in Figure 3A, B, D. Briefly, the dwell time τ was overlapped and averaged over every 2π interval, and the resulting average dwell time (related to adhesion strength) over a single 0-2π period was plotted in polar coordinates.

**Supplementary Figure S6.** Gallery of fluorescent footprint and footprint width distribution. (a) Gallery of fluorescent footprint of 11 cells. Solid vertical line marks an arbitrary start point of a repeating pattern. Dashed lines represent the first period of each track, which correspond to the circumference of each cell. Dashed horizontal line marks the mean circumference of the cell. Circle represent the size of an average cell. (b) Scaled illustration of cell deformation and the resulting in footprint width. (c) Histogram of footprint width (5.7 ± 1.3 μm, corresponding to an estimated cell contact area of 26 ± 12 μm^2^). The footprint width is defined as the 1/e width of the Gaussian fit to the cross-sectional intensity profiles along y, averaged for every 200-pixel interval along x.

**Supplementary Figure S7.** An example of image processing steps in the fluorescent footprinting assay. (a) Tiles of images were acquired automatically with 25% overlaps. The camera resolution is 512x512 pixel^2^. (b) Individual unflattened images were flattened by dividing each image by a normalized illumination profile constructed from the average of all unflattened images. The resulting flattened images tile is shown in (c). Strips of background images A (red dotted lines (a)) and B (blue dotted lines (c)) are taken over the same region in (a) and (c). (d) The intensity profile along *x* shows highly periodic patterns due to the uneven illumination profile before flattening (red). After flattening (blue) the periodicity is removed. (e) Normalized autocorrelation function of (d) showing the removal of periodicity of 512 pixels. (f) Image registration between adjacent image tiles allows stitching of individual frames in (c) into the final images, arrows indicated corresponding points in adjacent images (g) used for analysis.

**Supplementary Figure S8.** Control experiment demonstrating that cell rolling is due to P-selectin-specific interactions. (a) Illustration showing how P-selectin is immobilized on a PEG-5k passivated glass surface through neutravidin and biotinylated protein G or biotinylated TGT-Protein G. (b) Control experiments showing that only in the presence of all tethering proteins (neutravidin, Protein G/TGT-Protein G, and P-selectin-Fc) is cell rolling supported, and that the cell rolling is enabled solely by P-selectin interactions. The bottom three images display typical cell tracks over a 10 second period on surfaces prepared according to the colors. The long, straight tracks in the green and orange boxes correspond to cells that do not interact with surfaces and rapidly flow across the field of view. The short, undulating lines in the red box are tracks of rolling cells.

**Supplementary Figure S9.** Simulation of a random spatial distribution of microvilli on a rolling cell surface. (a) Snapshot of the surface distribution of microvilli (blue dots), and the ones in contact with surface (red dots). A microvilli density of 4 μm^-2^ was used based on previous studies ^1-3^ to generate the random distribution on the surface of a sphere. The green arrows indicate the direction of rolling. (b) Side view (x-z plane) of the distribution of surface-contacting microvilli around the circumference of the cell. (c) Number of surface-contacting microvilli as a function of angular position around the cell. (d) Polar representation of (c), where the radius represents the number of surface-contacting microvilli. (e) Footprint of microvilli on the surface (x-y plane) after the cell has completed two rotation cycles. (f) Simulated adhesion footprint and its normalized autocorrelation function from 8 rolling cycles. (g) The number of surface-contacting microvilli as a function of angular position around the cell for 200 simulated cells, each with a different random distribution of microvilli on its surface. (h) Histogram of (g), showing the variation in the number of surface-contacting microvilli between 130 and 200 at any given time.

**References**

1 Khismatullin, D. B. & Truskey, G. A. Leukocyte Rolling on P-Selectin: A Three-Dimensional Numerical Study of the Effect of Cytoplasmic Viscosity. *Biophysical journal* **102**, 1757-1766, doi:10.1016/j.bpj.2012.03.018 (2012).

2 Bruehl, R. E., Springer, T. A. & Bainton, D. F. Quantitation of L-selectin distribution on human leukocyte microvilli by immunogold labeling and electron microscopy. *J Histochem Cytochem* **44**, 835-844 (1996).

3 Majstoravich, S. *et al.* Lymphocyte microvilli are dynamic, actin-dependent structures that do not require Wiskott-Aldrich syndrome protein (WASp) for their morphology. *Blood* **104**, 1396-1403, doi:10.1182/blood-2004-02-0437 (2004).

**Supplementary Figure S1**


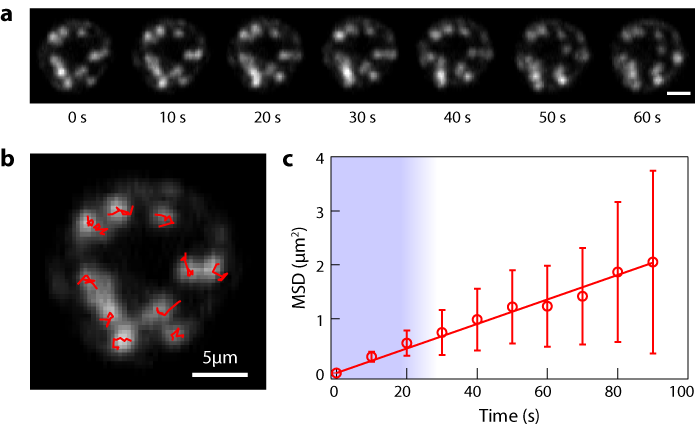


**Supplementary Figure S2**


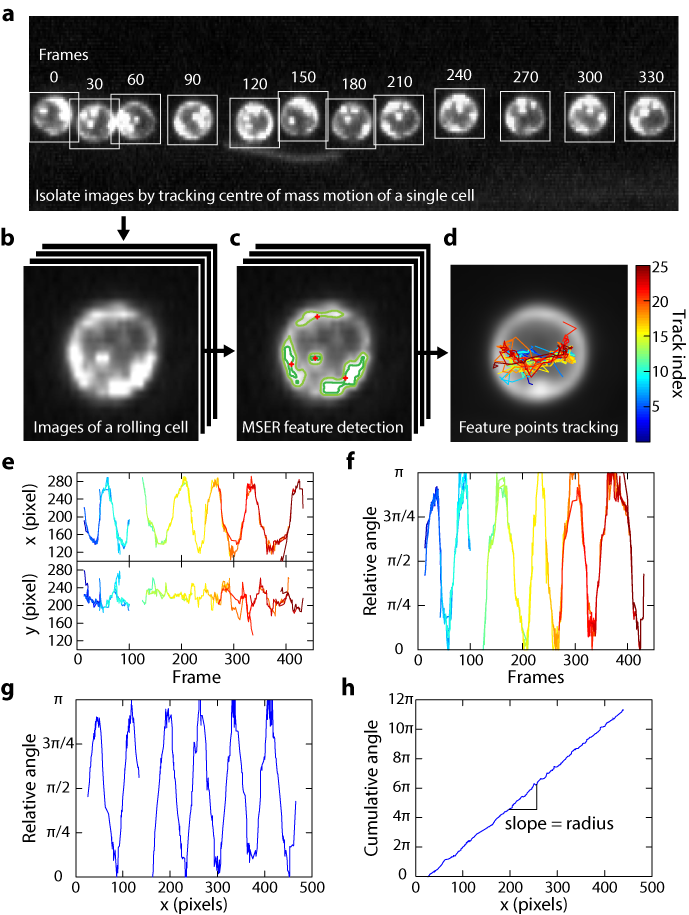


**Supplementary Figure S3**


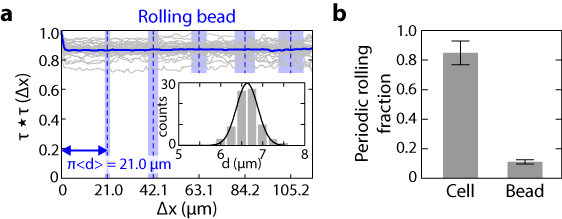


**Supplementary Figure S4**


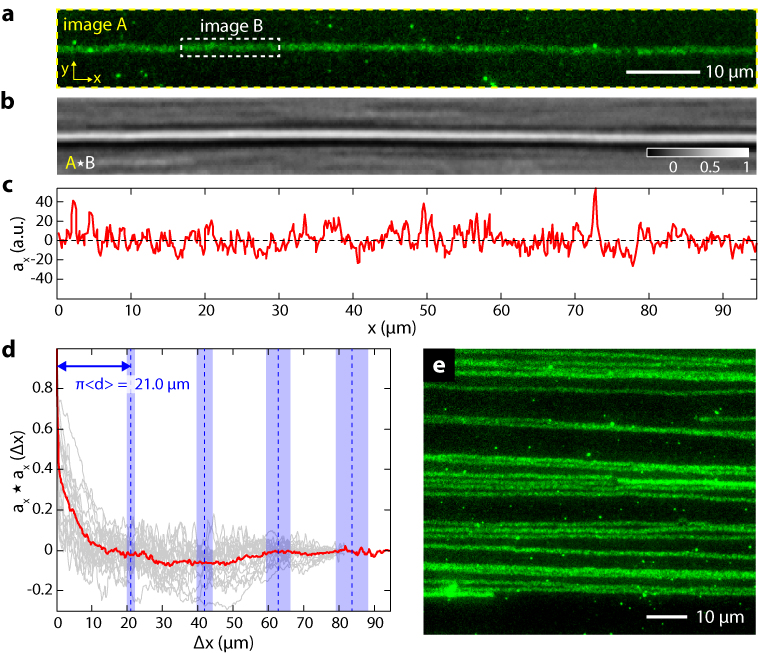


**Supplementary Figure S5**

**
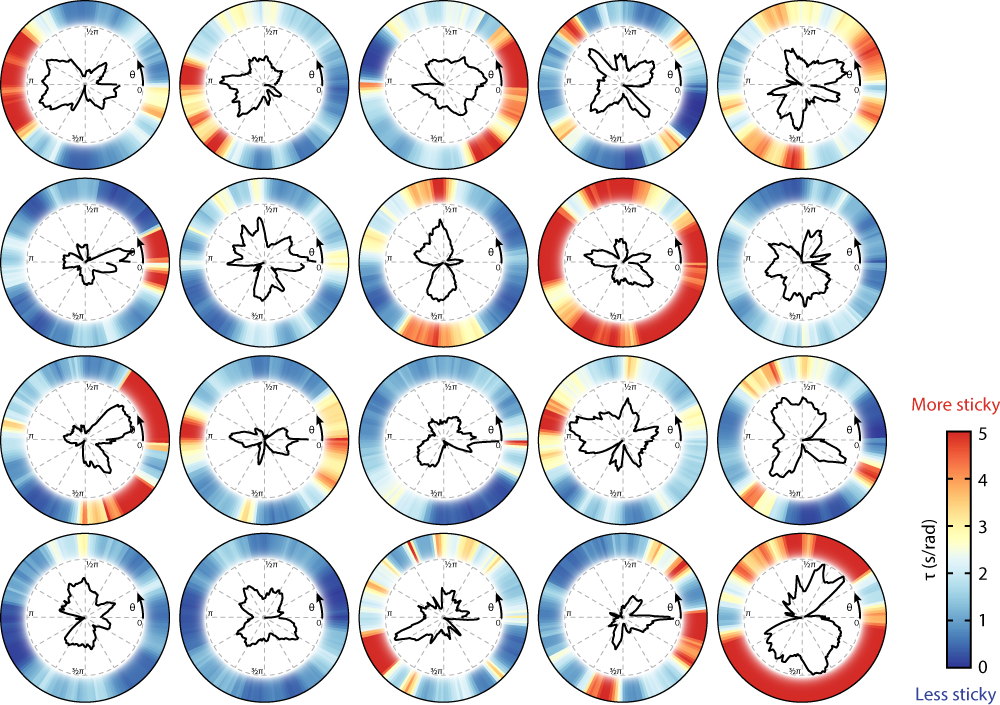
**

**Supplementary Figure S6**

**
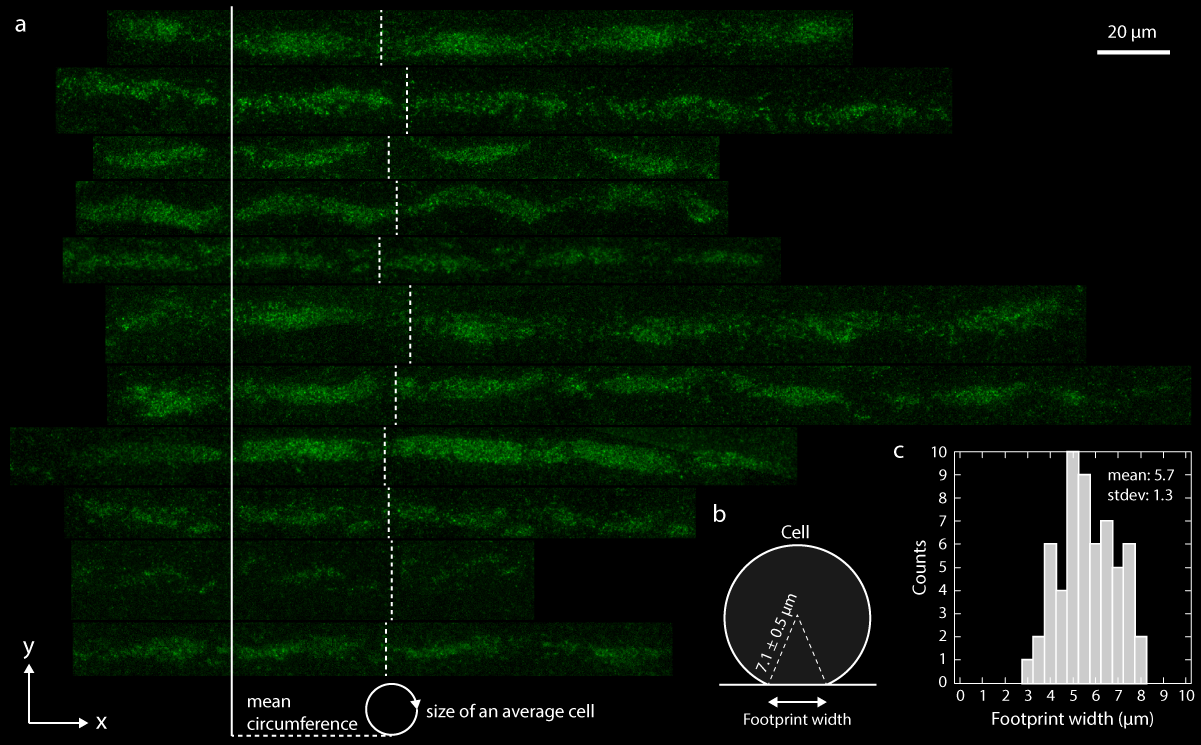
**

**Supplementary Figure S7**


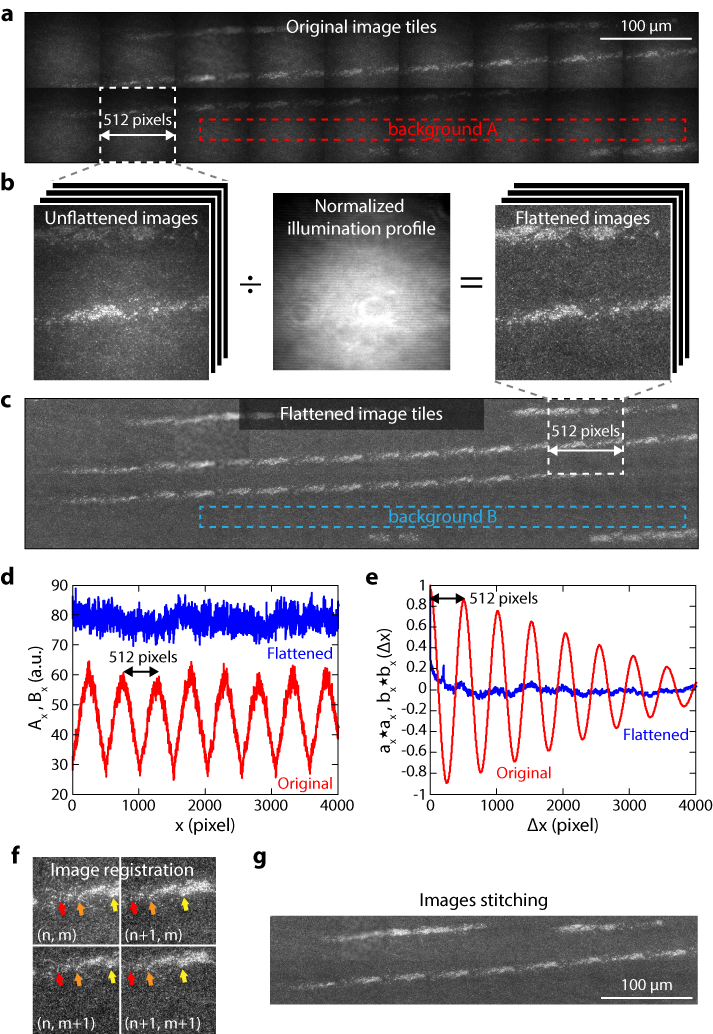


**Supplementary Figure S8**


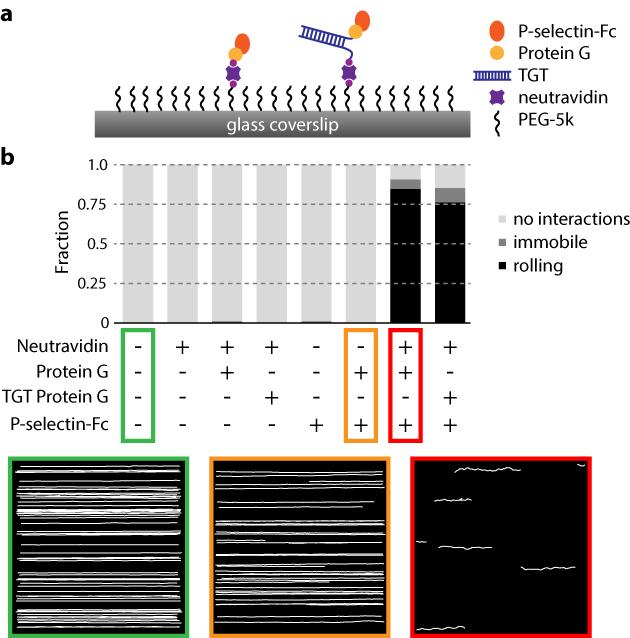


**Supplementary Figure S9**


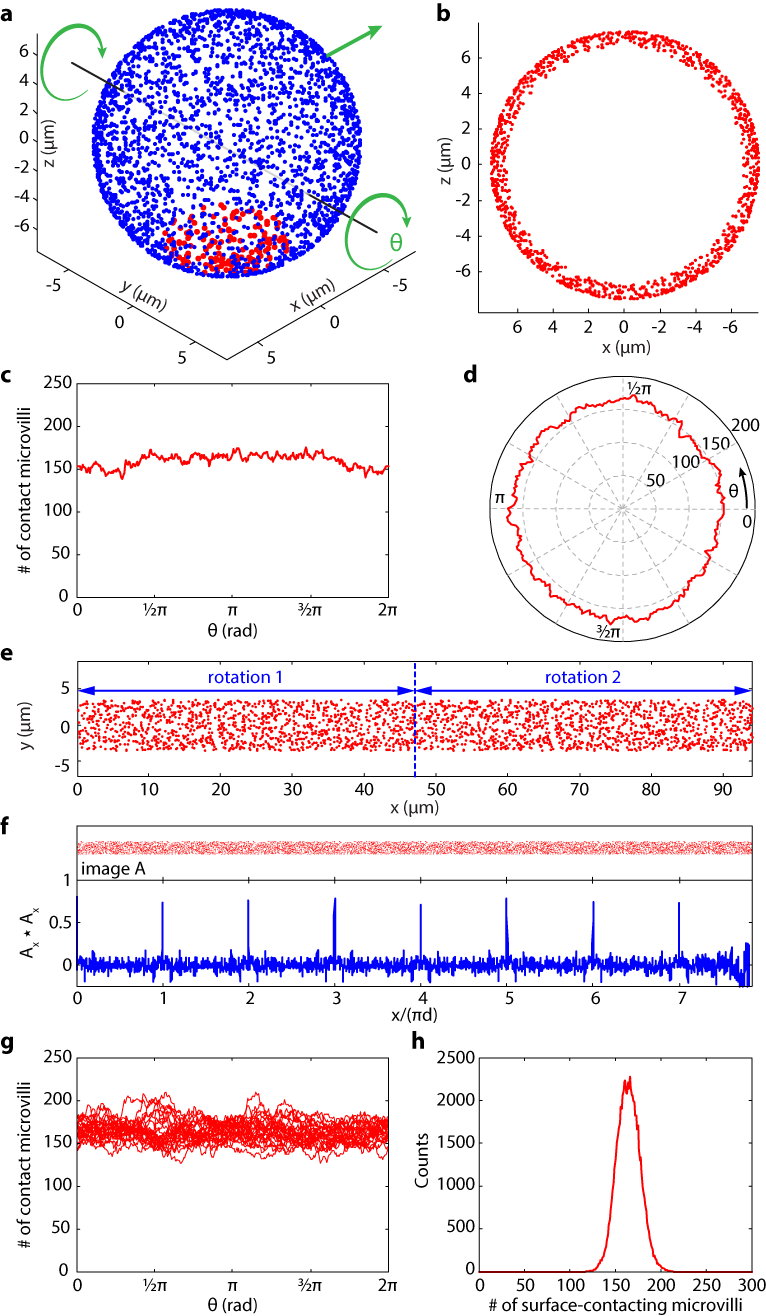

Supplement: Supplementary Information [file srep44502-s1.doc]
